# Impact of codon optimization on *vip3Aa11* gene expression and insecticidal efficacy in maize

**DOI:** 10.3389/fpls.2025.1579465

**Published:** 2025-05-13

**Authors:** Shengyan Li, Ning Wen, Wenjie Lv, Mingjun Zhang, Yinxiao Wang, Zhihong Lang

**Affiliations:** ^1^ Biotechnology Research Institute, Chinese Academy of Agricultural Sciences, Beijing, China; ^2^ College of Plant Protection, Jilin Agricultural University, Changchun, China; ^3^ National Nanfan Research Institute (Sanya), Chinese Academy of Agricultural Sciences, Sanya, Hainan, China

**Keywords:** codon optimization, *vip3Aa11* gene, transgenic maize, *Spodoptera frugiperda*, translation initiation, synonymous codon substitution

## Abstract

**Introduction:**

Codon optimization is critical for high expression of foreign genes in heterologous systems. The *vip3Aa11* gene from *Bacillus thuringiensis* is a promising candidate for controlling *Spodoptera frugiperda*.

**Methods and results:**

To develop insect-resistant maize, we designed two codon-optimized *vip3Aa11* variants (*vip3Aa11-m1* and *vip3Aa11-m2*) based on maize codon usage bias. Both recombinant proteins expressed in *Escherichia coli* exhibited high insecticidal activity. However, in transgenic maize, Vip3Aa11-m1 exhibited strong insecticidal activity against *Spodoptera frugiperda* and *Spodoptera exigua*, while Vip3Aa11-m2 lost activity despite identical amino acid sequences. RT-PCR analysis confirmed that both genes were transcribed correctly, but western blot results demonstrated a smaller product for *vip3Aa11-m2*, suggesting a translation-level alteration. Segment replacement and point mutation experiments in maize protoplasts demonstrated that the synonymous codon AAT (Asn) at the fourth amino acid position in *vip3Aa11-m2* was associated with the production of a truncated protein, suggesting that the AAT codon may influence the selection of the translation initiation site, potentially shifting it to a downstream ATG (Met) codon.

**Discussion:**

These findings not only reveal the critical role of codon context in translation initiation and protein integrity but also provide a novel strategy for optimizing foreign genes in crop improvement, particularly offering valuable insights for engineering insect-resistant maize using Bt genes.

## 1 Introduction

The development and commercialization of genetically modified (GM) crops expressing insecticidal toxin genes from *Bacillus thuringiensis* (Bt) have significantly contributed to increased food production, higher farmers’ incomes, and environmental protection over the past three decades (ISAAA, 2019). However, the sustainable application of Bt crops faces challenges, including the evolution of resistance in target pests and the emergence of invasive pests. To address these issues, ongoing research focuses on mining novel Bt genes, improving the efficient and stable expression of Bt genes in crops, and developing new insect-resistant events (Kumar et al., 2020). Several approaches have been employed to increase Bt toxin accumulation in GM plants, such as using strong promoters and enhancer elements, optimizing codon usage to match the host’s codon preference, and utilizing fusion tags (e.g., MBP, GST) to enhance protein solubility and stability (Streatfield, 2007). Among these, codon optimization has become the most widely adopted method due to the limited availability of promoters and regulatory elements, as well as safety concerns related to the presence of non-functional fusion proteins in GM crops.

Codon usage bias, a universal biological phenomenon, influences gene expression through processes such as RNA processing, protein translation, and protein folding (Hanson and Coller, 2018; Parvathy et al., 2022). Codon optimization based on host codon usage bias is an effective strategy for achieving high expression of Bt genes in plants. For instance, it has been demonstrated that codon optimization of the *cry1Ab* gene resulted in a 100-fold increase in Cry1Ab protein expression in tobacco and tomato compared to the wild-type gene (Perlak et al., 1991). With advances in genetic sequencing and bioinformatics, a variety of algorithms and software tools-such as genetic algorithms, the Ribotree Monte Carlo tree search method, dynamic programming, and Codon Optimization OnLine (COOL)-have been developed to design codon-optimized synthetic genes with desired characteristics (Chin et al., 2014; Blazej et al., 2018; Taneda and Asai, 2020; Leppek et al., 2022). However, codon optimization may introduce unintended risks. Studies have shown that changes in synonymous codons can alter protein conformation and stability, potentially disrupting proper folding and impairing function, while also interfering with critical post-translational modifications such as phosphorylation and glycosylation (Liu, 2020; Moss et al., 2024). Furthermore, the effectiveness of codon optimization is not always predictable, as it depends on the compatibility between the optimized sequence and the host organism’s regulatory mechanisms.

The fall armyworm (FAW, *Spodoptera frugiperda*), a highly destructive pest native to the Americas, has emerged as a global threat to crop production. Initially, the FAW-resistant transgenic maize TC1507, harboring Bt *cry1F* gene, achieved over 99% field control efficacy (Siebert et al., 2008). However, prolonged and extensive use of TC1507 has led to a significant decline in FAW’s susceptibility to Cry1F protein, with resistant populations now found in field conditions (Storer et al., 2010; Farias et al., 2014). Vegetative insecticidal proteins (Vips), particularly those from the Vip3A subfamily, demonstrate strong activity against *Spodoptera* species that exhibit low susceptibility to Cry proteins (Chakroun et al., 2016). For instance, Vip3Aa19 and Vip3Aa20 have shown high efficacy against FAW and have been successfully introduced into GM maize. To date, no field resistance of FAW to Vip3Aa protein has been reported (Yang et al., 2019).

The *vip3Aa11 gene*, a member of the Vip3A subfamily cloned from the Bt strain C9, demonstrates high insecticidal activity against various *lepidopteran* pests, including the cotton bollworm (*Helicoverpa armigera*), the beet armyworm (BAW, *Spodoptera exigua*) and FAW (Liu et al., 2004; Ma et al., 2024). To develop transgenic maize with high Vip3Aa11 expression, we optimized the *vip3Aa11* gene sequence based on maize codon usage bias, generating two variants: *vip3Aa11-m1* and *vip3Aa11-m2*. Although both variants showed high expression in maize, *vip3Aa11-m2* unexpectedly lost insecticidal activity despite its higher expression level. Through molecular experiments, we discovered that the AAT codon at the fourth amino acid position (N4) in *vip3Aa11-m2* caused a shift in the translation initiation site, resulting in a truncated protein with no insecticidal activity. This finding underscores the importance of avoiding the AAT codon at the N4 site during genetic engineering to prevent protein inactivation.

## 2 Materials and methods

### 2.1 Codon optimization and construction of vectors

The full-length nucleotide sequences of *vip3Aa11* (AY489126.1) gene was provided and authorized by Prof. Jie Zhang from Institute Plant Protection of CAAS. The *vip3Aa11* gene was optimized and modified according to OptimumGene, an algorithm developed by the GenScript Biotech Corporation (Nanjing, China). The codon-optimized *vip3Aa11-m1* and *vip3Aa11-m2* genes were synthesized by GenScript Biotech Corporation. The *vip3Aa11-m1* and *vip3Aa11-m2* genes were ligated into *Bam*H I/*Xho* I-digested pET32a by seamless assembly cloning (A14603, Thermo Fisher Scientific, Waltham, MA, USA) to generate the prokaryotic expression vectors: pVm1 and pVm2, respectively. The nucleotide sequences of *vip3Aa19* and *vip3Aa20* genes were modified and point-specific mutated from *vip3Aa11-m2* gene, and ligated into *Bam*H I/*Xho* I-digested pET32a to generate pV19 and pV20 vectors. The *vip3Aa11-m1* gene and glyphosate resistance gene *cp4-epsps* were ligated into pDONR221 P1-P5r (V011824, Invitrogen, Carlsbad, CA, USA) and pDONR221 P5-P2 (V011821, Invitrogen, Carlsbad, CA, USA) vectors by Gateway BP reaction (11789, Invitrogen, Carlsbad, CA, USA), with maize *Ubiquitin* promoter and *NOS* terminator, respectively. Then, the *vip3Aa11-m1* and *cp4-epsps* cassettes were ligated into binary vector pPZP200-R1R2 which was modified from pPZP200 by Gateway LR reaction (11791, Invitrogen, Carlsbad, CA, USA) to generate the maize transformation vector pVP1. The *vip3Aa11-m2* cassette, with maize *Ubiquitin* promoter and *NOS* terminator, was ligated into binary vector pCAMBIA3300 by seamless assembly cloning (A14603, Thermo Fisher Scientific, Waltham, MA, USA) to generate the maize transformation vector pVP2. The *bar* gene was used as selection marker gene.

### 2.2 Prokaryotic expression and purification of Vip3Aa proteins

The pVm1, pVm2, pV19, and pV20 vectors were transformed into *Escherichia coli* Rosetta (DE3) cells. The induced expression of Vip3Aa proteins in *E.coli* Rosetta (DE3) cells were performed as previously described (Li et al., 2018). The cell pellet was resuspended in BugBuster Master Mix (71456, Merck, Darmstadt, Germany) and lysed for 20 minutes on ice. The supernatant was collected by centrifugation at 13,000 g for 15 min at 4°C, and the pellet was resuspended with phosphate-buffered saline (PBS) containing 0.1% (*v/v*) Tween-20. These four Vip3Aa proteins in supernatant were purified by His-Tagged Protein Purification Kit (CW0894S, CWBIO, Beijing, China) following the manufacturer’s protocols. The purified protein concentrations were detected by Bradford Assay.

### 2.3 Immunoblot analysis of Vip3Aa proteins

The Vip3Aa protein (AA1611) was purchased from YouLong Biotech, Shanghai, China, and used as a positive control. The proteins were separated on 10% SDS-polyacrylamide gels and blotted onto a PVDF membrane (IPVH00010, Merck, Kenilworth, NJ, USA) using an electrophoretic transfer (Bio-Rad, Hercules, CA, USA). The PVDF membrane was blocked with 5% (*w*/*v*) skim milk in Tris-buffered saline containing 0.1% (*v*/*v*) Tween-20 (TBST) for 1 hour at room temperature to prevent nonspecific binding. Subsequently, the membrane was incubated overnight at 4°C with primary antibody diluted in TBST containing 5% (*w*/*v*) skim milk. After three 10-minute washes with TBST, the membrane was incubated with the secondary antibody diluted in TBST for 1 hour at room temperature. Anti-Vip3Aa (AA1624, YouLong Biotech, Shanghai, China) primary antibody was used at a 1:5,000 dilution, and an HRP-conjugated goat anti-mouse (CW0102, CWBIO, Beijing, China) secondary antibody was used at a 1:10,000 dilution. Protein signals were detected in an Amersham Imager 600 (GE Healthcare, Chicago, IL, USA) using an eECL Western blot kit (CW0049, CWBIO, Beijing, China).

### 2.4 Maize transformation

The maize expression vector pVP1 and pVP2 were transformed into B104 maize immature embryos by *Agrobacterium* (*Agrobacterium tumefaciens* strain EHA105)-mediated transformation as previously described (Frame et al., 2002). *Agrobacterium tumefaciens* was cultured on YEP medium supplemented with 50 mg/L rifampicin and 50 mg/L kanamycin for 3d at 19°C. Bacteria cells were scraped from the plate and resuspended in 5 mL of liquid infection medium containing 100 μM acetosyringone (AS) in a 50 mL tube. The suspension was gently agitated at 75 rpm for 2-4 h at room temperature until reaching an OD_550_ of 0.3-0.4. Embryos were immersed in the bacterial suspension by gently inverting the tube 20 times. Following a 5-minute incubation period in the dark, the embryos were transferred to the cocultivation medium and cultured at 20°C in the darkness for 3d. Subsequently the maize embryos were subjected to selection. Positive calli and regenerated plantlets were selected on Murashige and Skoog medium containing glyphosate or glufosinate (0.2 g L^−1^). All plant tissue culture reagents were purchased from PhytoTechnology Laboratories (Shawnee Mission, KS, USA).

### 2.5 PCR, RT-PCR, and ELISA analysis of transgenic maize

The genomic DNA was isolated from fresh maize leaf tissues using the CTAB method (Porebski et al., 1997). The PCR primers of the *vip3Aa11-m1*, *vip3Aa11-m2*, *cp4-epsps* and *bar* genes were used to confirm the transgenic maize plants, as detailed in **Supplementary Table S1**. The PCR products were visualized with gel-red and documented on G:BOX BioImaging systems (SYNGENE, Cambridge, UK). The total RNA was extracted from fresh maize leaf tissues using TRIzol reagent (R401, Vazyme, Nanjing, China). The cDNA was obtained by RevertAid First Strand cDNA Synthesis Kit (K1622, Thermo Fisher Scientific, Waltham, MA, USA) following the manufacturer’s protocols. The primers vip3Aa11-m2-F2/R2 were used to clone the full length of *vip3Aa11-m2* gene from the cDNA of pVP2 transgenic maize plants. Enzyme-linked immunosorbent assay (ELISA) kits for Vip3Aa (AA1641, YouLong Biotech, Shanghai, China) was used to detect the amounts of Vip3Aa11 protein in the transgenic maize leaves following the manufacturer’s protocol. The optical density (OD) of the samples was measured at 450 nm and 630 nm using a BioTek Elx808 plate reader (BioTek Instruments, Winooski, Vermont, USA).

### 2.6 Insect bioassays

The evaluation of insecticidal activity of *E.coli*-expressed Vip3Aa proteins on FAW (neonate larvae) were conducted on fresh leaf discs by leaf dip bioassays (Tabashnik et al., 1993). The neonate larvae of FAW were purchased from MeiYan (Beijing) Agricultural Technology Co., Ltd. (Beijing, China). Disks (1 cm diameter) were cut from fully expanded leaves of B104 grown in the greenhouse. Disks were dipped for 20 minutes in double-distilled water containing 0.1% (*v/v*) Triton X-100. Using a brush, the surface of the disks was gently scrubbed to remove dust and wax, and then the disks were dried with absorbent paper. The purified Vip3Aa proteins were diluted with phosphate-buffered saline (PBS) containing 0.1% (*v/v*) Triton X-100 into six concentration gradients: 0.05 μg/mL, 0.1 μg/mL, 1 μg/mL, 10 μg/mL, 20 μg/mL, and 30 μg/mL. The clean disks were dipped for 20 seconds in Vip3Aa protein dilutions and hung vertically to air dry at 23°C. The disks, which were dipped in PBS containing 0.1% (*v/v*) Triton X-100, were used as negative control. Each disk was then placed into a well of 24-well plate (3524, Corning, Kennebunk, ME, USA). One neonate larva was placed on each disk. We performed three biological replicates with each treatment. All plates were incubated in a rearing room at 70-80% relative humidity, 26°C, and a photoperiod of 16 h light/8 h dark. The number of living and dead larvae was recorded after 7 days.

The resistance of transgenic maize plants to FAW were evaluated using leaves by 24-well plate assay. The leaves of transgenic maize plants were cut into 1-cm segments and placed into each well of a 24-well plate (3524, Corning, Kennebunk, ME, USA), using B104 leaves as control. One neonate larva of FAW was placed into each well. The plates were placed in the rearing room at 70-80% relative humidity, 26°C, and a photoperiod of 16 h light/8 h dark. Each treatment contained 3 biological replications. The number of living and dead larvae was recorded every day for 7 days.

The eggs of beet armyworm (BAW, *Spodoptera exigua*) were kindly supplied by Prof. Wei Guo from the Graduate School of CAAS. The eggs were maintained in the dark at 70-80% relative humidity, 28°C in the rearing room for incubation. The neonate BAW larvae were used for maize plants inoculation. The BAW bioassay of transgenic maize plants in greenhouse were performed as previously described (Zheng et al., 2000). The two-week-old maize plants were put into transparent hard plastic cage. Ten neonate BAW larvae were inoculated with a paintbrush on the base of each plant. Each treatment contained five maize plants. The experiment was carried out in the greenhouse at 26°C and a photoperiod of 16 h light/8 h dark. Three days after inoculation the number of surviving BAW larvae per plant were recorded. Mortality rates are presented as the proportion of dead larvae to total larvae (%).

### 2.7 Maize protoplast transformation

The variants of *vip3Aa11* gene were amplified by overlap extension PCR with the primers listed in **Supplementary Table S1**. The PCR products were then ligated into *Spe* I/*Kpn* I-digested p2GW7 by seamless assembly cloning (A14603, Thermo Fisher Scientific, Waltham, MA, USA), generating a series of constructs driven by the maize *Ubiquitin* promoter and terminated by the *NOS* terminator. The middle of the second euphylla from two-week-old maize yellow seedling was used for the protoplast isolation as described previously (Li et al., 2015). The series of constructs were transfected into the maize protoplasts by using 30% (*w/v*) PEG-calcium transfection solution, respectively. After 12 h of culture at 23°C in dark, the protoplasts were harvested and added 100 μL Cell Culture Lysis Reagent (CCLR) (E1531, Promega, Madison, USA) to complete lysis. The supernatant was transferred to a new tube for the next step of immunoblot analysis.

### 2.8 Statistical analysis

All datasets were initially processed using Microsoft Excel 2021 (Microsoft Corporation, Redmond, WA, USA). Data normality and homogeneity of variance were assessed using GraphPad Prism 9.4 (GraphPad Software, Boston, MA, USA) and IBM SPSS Statistics 26 (IBM Corporation, Armonk, NY, USA). Appropriate statistical tests were subsequently selected based on these evaluations for significance analysis.

## 3 Results

### 3.1 Codon optimization of the *vip3Aa11* gene

**Figure 1:**
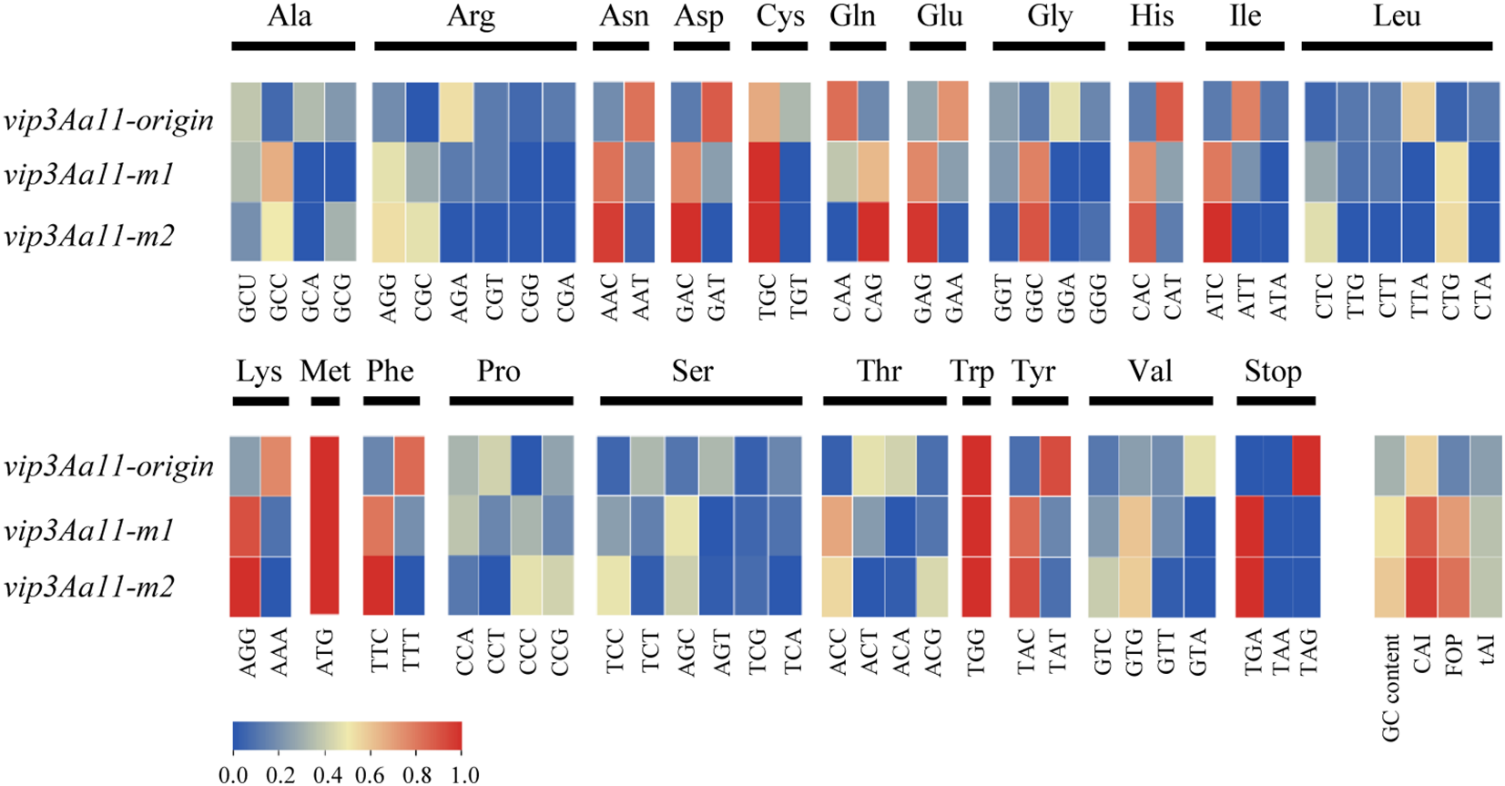
Comparison of codon frequency; GC content; CAI; FOP and tAI among the original *vip3Aa11*; *vip3Aa11-m1* and *vip3Aa11-m2*. CAI, Codon Adaptation Index; FOP, Frequency of Optimal Codons; tAI, tRNA Adaptation Index.

To achieve high expression of the Vip3Aa11 protein in maize, we optimized the *vip3Aa11* gene based on maize codon usage bias. Two codon optimization strategies were employed. The first one was designed by maintaining codon diversity while replacing rare codons in maize, which was designated *vip3Aa11-m1*. And the second one was designed by selecting the optimal codon for each amino acid based on the maize genome, and was designated *vip3Aa11-m2*. Comparison of the codon frequency per corresponding amino acid among *vip3Aa11-origin*, *vip3Aa11-m1*, and *vip3Aa11-m2* was shown in **Figure 1**. The codon adaptation index (CAI) and frequency of optimal codons (FOP) of the *vip3Aa11-origin* gene for maize were 0.56 and 0.15, with GC contents of 31%. After optimization, the CAIs of the *vip3Aa11-m1* and *vip3Aa11-m2* genes increased to 0.88 and 0.96, respectively, while their FOPs rose to 0.71 and 0.82, with their GC contents at 52% and 59% (**Figure 1**).

### 3.2 The prokaryotically expressed Vip3Aa11-m1 and Vip3Aa11-m2 proteins exhibited identical insecticidal activity against the FAW

**Figure 2:**
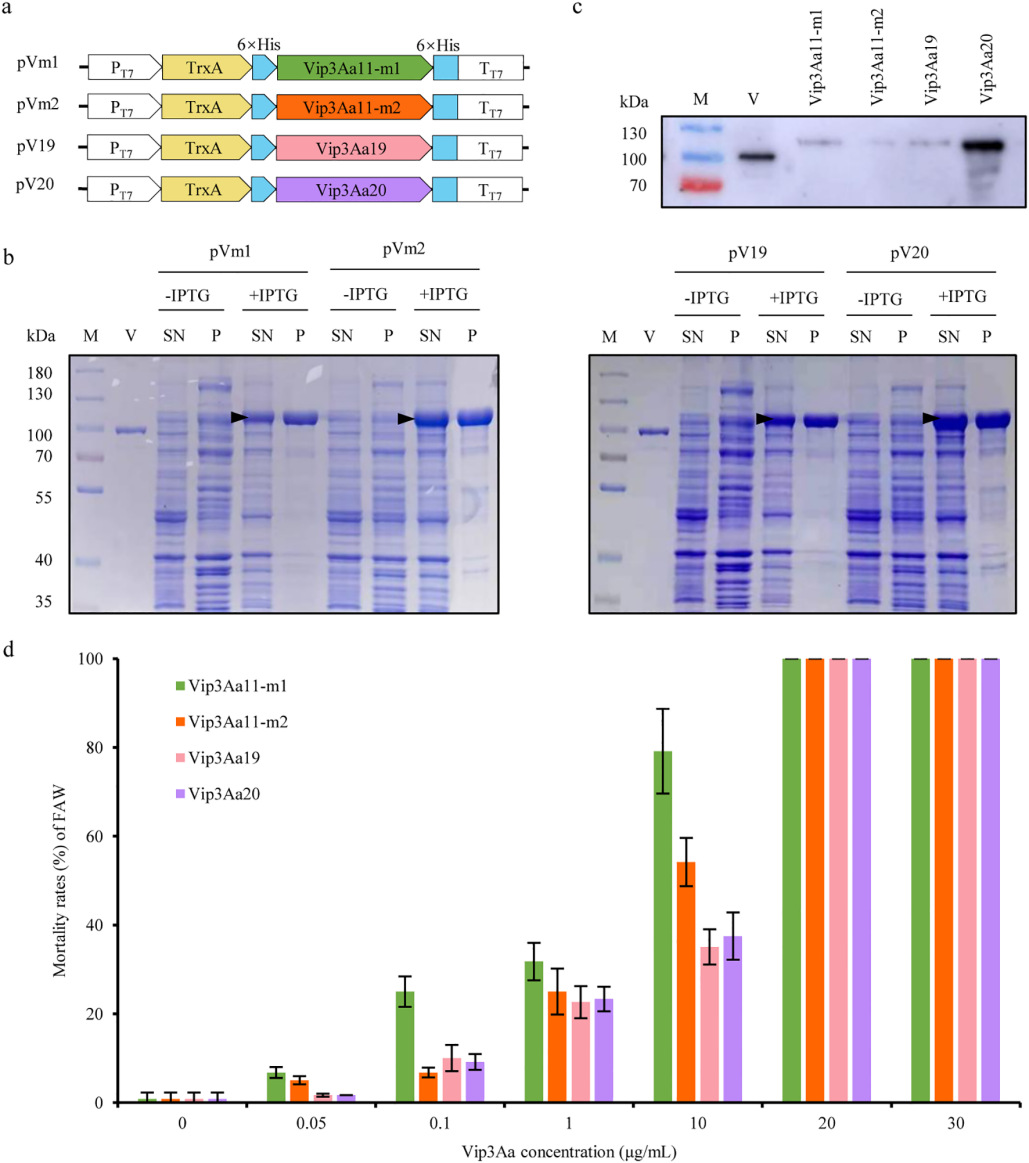
Prokaryotic expression of Vip3Aa11-m1 and Vip3Aa11-m2 proteins and analysis of their insecticidal activity against fall armyworm (FAW). **(a)** Schematic diagram of the prokaryotic expression vectors. **(b)** SDS-PAGE analysis of Vip3Aa protein expression before and after IPTG induction. M: PageRuler™ prestained protein ladder (10-180 kDa); V: commercial Vip3Aa protein (AA1611); SN: supernatant; P: pellet. The black triangle indicates the position of the Vip3Aa fusion protein. **(c)** Western blot analysis of the purified Vip3Aa11-m1, Vip3Aa11-m2, Vip3Aa19 and Vip3Aa20 proteins. The commercial Vip3Aa protein (V) was used as a positive control. M: PageRuler™ prestained protein ladder (10-180 kDa). **(d)** Leaf dip bioassay of the insecticidal activity of *E.coli*-expressed Vip3Aa proteins against FAW. The leaf disks dipped in PBS buffer containing 0.1% (*v/v*) Triton X-100 served as negative control. Data represent means ± SD (n=3 biological replicates).

In order to assess the insecticidal activity of Vip3Aa11-m1 and Vip3Aa11-m2 proteins against the FAW, prokaryotic expression vectors, namely pVm1 and pVm2, were constructed using the optimized *vip3Aa11-m1* and *vip3Aa11-m2* genes, respectively. Subsequently, these vectors were transformed into *E. coli* Rosetta (DE3) for the induction of Vip3Aa11 protein expression, with *vip3Aa19* (pV19) and *vip3Aa20* (pV20) as controls (**Figure 2A**). The expected size of Vip3Aa11-m1, Vip3Aa11-m2, Vip3Aa19, and Vip3Aa20 proteins, including the fusion tag, were approximately 104 kDa. The SDS-PAGE results demonstrated that the target proteins of the expected sizes were expressed in the supernatant of the lysed bacterial cells after IPTG induction (**Figure 2B**). Immunoblotting results indicated that a single band was detected from each of the four purified Vip3Aa proteins (**Figure 2C**). The four purified Vip3Aa proteins were diluted with phosphate-buffered saline (PBS) containing 0.1% (*v/v*) Triton X-100 into six concentration gradients of 0.05 μg/mL, 0.1 μg/mL, 1 μg/mL, 10 μg/mL, 20 μg/mL, and 30 μg/mL, which were utilized for bioassays. The experimental results indicated that as the concentration of the four Vip3Aa proteins increased, the mortality rates of FAW larvae increased correspondingly. When the Vip3Aa protein concentration reached 20 μg/mL, the mortality rates of the FAW larvae for all four Vip3Aa proteins were 100% (**Figure 2D**). This indicates that the Vip3Aa11-m1 and Vip3Aa11-m2 proteins possess high insecticidal activity against the FAW comparable to that of Vip3Aa19 and Vip3Aa20.

### 3.3 Expression of Vip3Aa11 protein higher in VP2 transgenic maize than in VP1 transgenic maize

**Figure 3:**
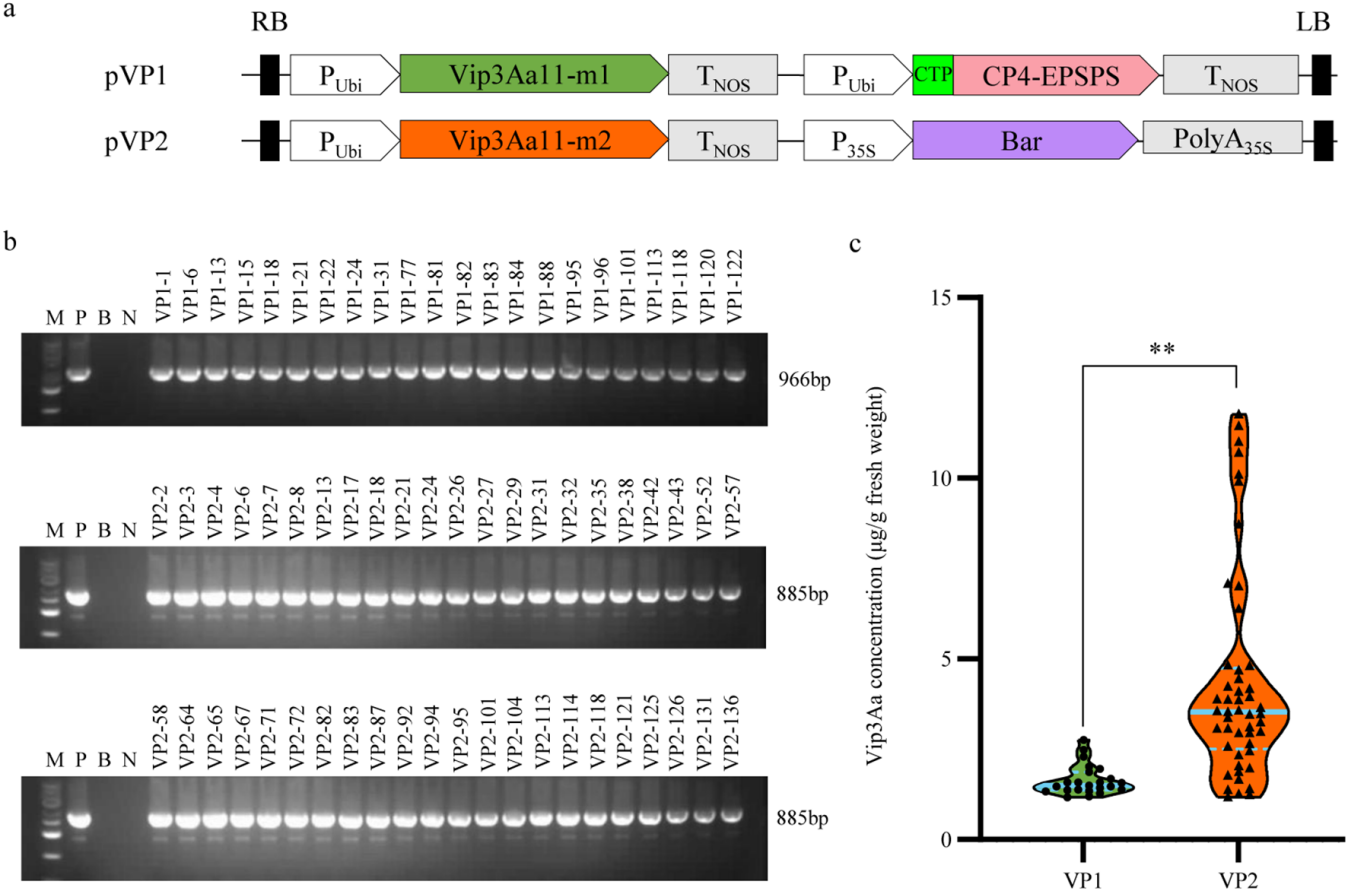
PCR screening of the regenerated maize plants and Enzyme-linked immunosorbent assay (ELISA) of Vip3Aa11 expression in transgenic maize plants. **(a)** Schematic diagram of the maize genetic transformation constructs pVP1 and pVP2. The glyphosate resistance gene *cp4-epsps* and glufosinate resistance gene *bar* were used as selected marker genes for maize transformation, respectively. **(b)** PCR analysis of the *vip3Aa11-m1* and *vip3Aa11-m2* genes in corresponding transgenic maize plants. M: 5kb Plus DNA Marker (100-5000 bp); P: positive control pVP1 or pVP2 vector; B: blank; N: wild-type B104 plants. **(c)** Detection of Vip3Aa11 protein expression in transgenic maize plants by ELISA. **P<0.01, determined using Welch’s *t-test*.

In prokaryotic expression studies, both Vip3Aa11-m1 and Vip3Aa11-m2 protein demonstrated significant insecticidal activity against the FAW. To develop FAW-resistant transgenic maize plants, we constructed two distinct maize transformation vectors: pVP1, harboring the *vip3Aa11-m1* and *cp4-epsps* genes, and pVP2, containing the *vip3Aa11-m2* and *bar* gene. (**Figure 3A**). Two transformation vectors, pVP1 and pVP2, were transformed into maize via *Agrobacterium*-mediated transformation. Subsequently, a total of 136 and 120 regenerated maize plants were obtained from the pVP1 and pVP2 vectors, respectively. Employing polymerase chain reaction (PCR) and Enzyme-linked immunosorbent assay (ELISA) for screening, we identified 23 VP1 lines and 49 VP2 lines in which the Vip3Aa11 protein expression exceeded 1 µg/g Fresh Weight (FW) (**Figures 3B, C**). The maximum expression level of Vip3Aa11 in VP1 transformants and VP2 transformants were 2.76 µg/g FW and 11.65 µg/g FW, respectively (**Figure 3C**). Welch’s *t-test* analysis revealed that the *vip3Aa11-m2* gene, characterized by a higher Codon Adaptation Index (CAI), led to significantly higher protein expression in maize compared to the *vip3Aa11-m1* gene (**Figure 3C**). This finding suggested codon optimization can effectively enhance protein expression within host cells, highlighting the importance of codon usage in transgenic plant development for improved pest resistance.

### 3.4 High expression of Vip3Aa11-m2 protein but loss insecticidal activity in maize

**Figure 4:**
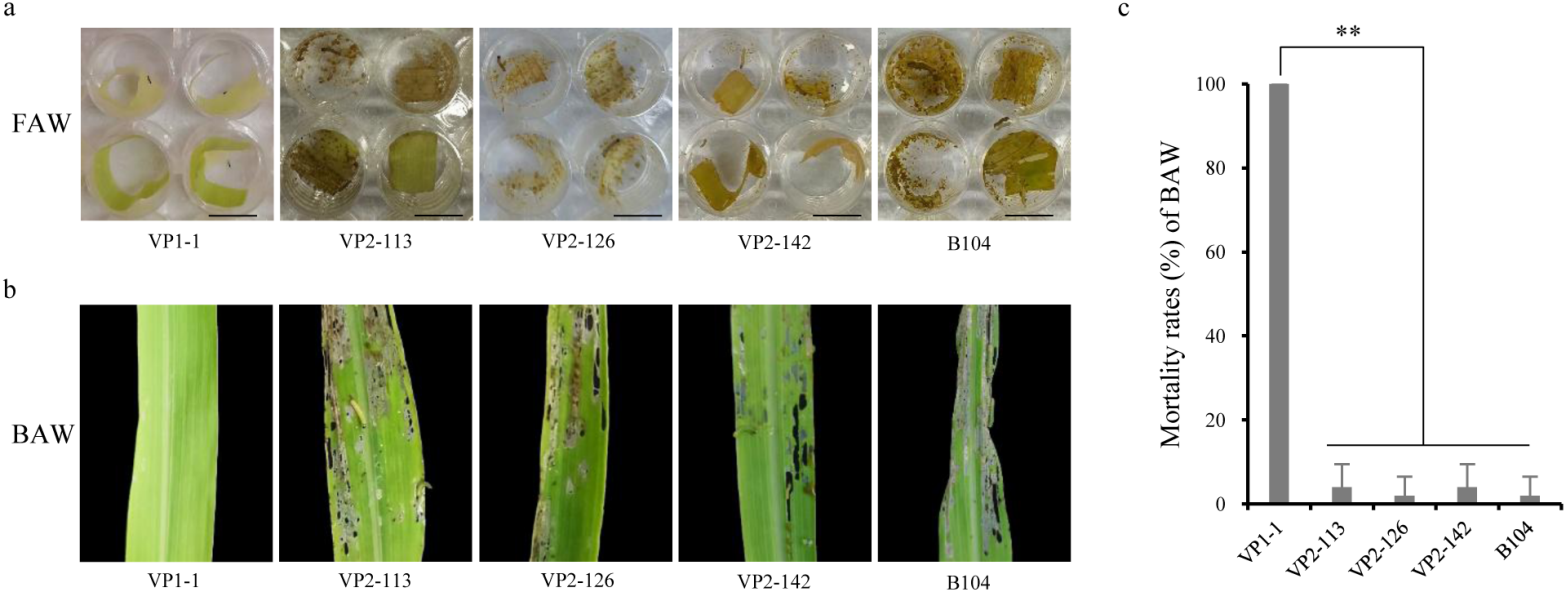
Laboratory bioassays of VP1 and VP2 transgenic maize plants with fall armyworm (FAW) and beet armyworm (BAW). **(a)** The appearance of wild-type B104 and transgenic maize leaves after insect bioassays with FAW. Photographs were taken after 5 days of infestation. Scale bar=1 cm. **(b)** The appearance of wild-type B104 and transgenic maize leaves after insect bioassays with BAW. Photographs were taken after 3 days of infestation. **(c)** The mortality rates of BAW larvae feeding on the leaves of wild-type B104 and VP1-1, VP2-113, VP2-126, VP2-142 transgenic maize plants. Data represent means ± SD (n=5 biological replicates). **P<0.01, determined using one-way ANOVA.

Through ELISA analysis, it was found that the expression of Vip3Aa11-m2 protein in VP2 transgenic maize population was significantly higher than that of Vip3Aa11-m1 protein in VP1 transgenic maize population. Subsequently, bioassays were conducted using 23 VP1 and 49 VP2 transgenic maize lines. Laboratory bioassay results showed that all 23 VP1 transgenic maize lines exhibited high insecticidal activity against FAW, with all FAW larvae dying within 5 days infestation. In contrast, the leaves of the 49 VP2 transgenic maize lines were severely damaged, and the mortality rates of FAW larvae were not significantly different from the wild-type leaves (**Figure 4A** and **Supplementary Table S2**). These results suggest that the 49 VP2 transgenic maize lines lacked insecticidal activity against FAW.

To determine whether the observed inactivity was specific to FAW or extended to other target pests, we selected beet armyworm (BAW, *Spodoptera exigua*), another pest from the family Noctuidae, for bioassay. One VP1 transgenic maize line (VP1-1) and three VP2 transgenic maize lines (VP2-113, VP2-126, VP2-142) were subjected to BAW bioassay. The results were consistent with those observed in FAW bioassays. Three days after infestation, the leaves of VP1-1 remained undamaged and all BAW larvae had died. In contrast, the leaves of VP2-113, VP2-126, and VP2-142, along well as those of B104, were all severely damaged by BAW larvae (**Figure 4B**). Statistical analysis showed that the BAW mortality rates of VP2-113, VP2-126, and VP2-142 were not significantly different from that of B104, but were highly significantly different from that of VP1-1 (**Figure 4C**). These finding suggest that the Vip3Aa11-m2 protein in VP2 transgenic maize lines lacked insecticidal activity to BAW. Consequently, these results indicate that both codon-optimized *vip3Aa11-m1* and *vip3Aa11-m2* genes enabled high express of Vip3Aa11 proteins in maize. However, despite its higher expression level in maize, the Vip3Aa11-m2 protein completely lacked insecticidal activity.

### 3.5 The loss of insecticidal activity of Vip3Aa11-m2 protein in transgenic maize is caused by a truncated protein form

**Figure 5:**
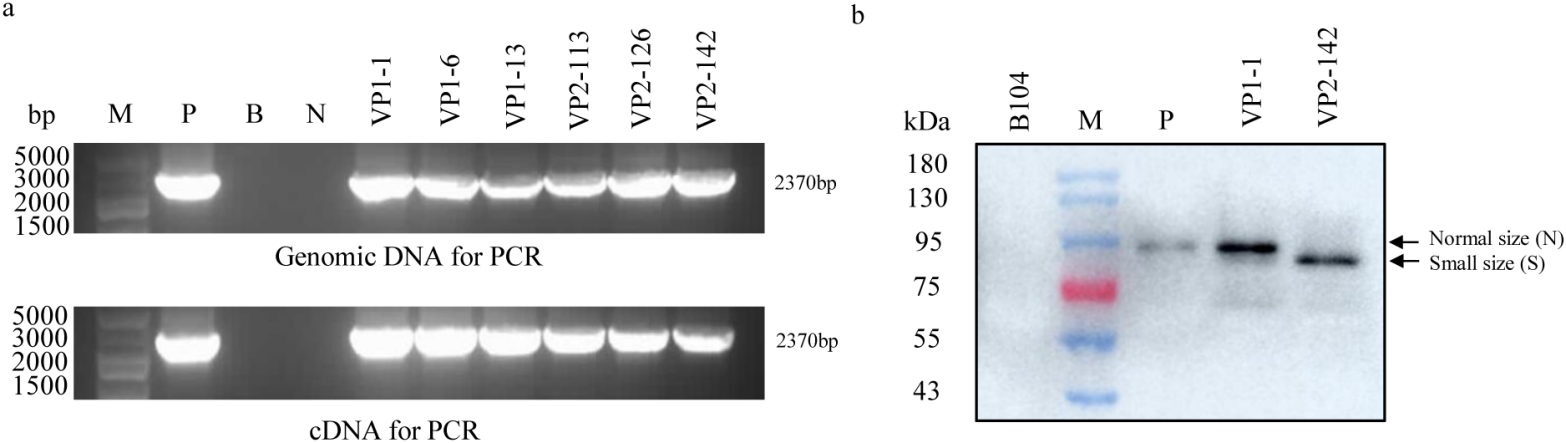
PCR, RT-PCR, and immunoblot analysis of the size of *vip3Aa11-m2* gene and Vip3Aa11-m2 protein in VP2 transgenic maize plants. **(a)** The full-length *vip3Aa11-m1* and *vip3Aa11-m2* genes were cloned from the genomic DNA and cDNA of VP1 and VP2 transgenic maize plants using PCR and RT-PCR, respectively. M: 5kb Plus DNA Marker (100-5000 bp); P: positive control pVP2 vector; B: blank; N: wild-type B104 plants. **(b)** Immunoblot analysis of the sizes of Vip3Aa11-m1 and Vip3Aa11-m2 proteins in VP1 and VP2 transgenic maize plants. M: prestained protein ladder (10-180 kDa). P: commercial Vip3Aa protein.

To elucidate the underlying cause of the loss of insecticidal activity of the Vip3Aa11-m2 protein in VP2 transgenic maize, we conducted a comprehensive analysis the *vip3Aa11-m2* gene at the genomic, transcriptional, and translational levels. Using PCR and RT-PCR, we successfully isolated the full-length *vip3Aa11-m2* gene from both the genomic DNA and cDNA of VP2 transgenic maize plants (**Figure 5A**). Subsequent Sanger sequencing and BLAST analysis confirmed that no mutations were present in the *vip3Aa11-m2* gene at both the genomic DNA and mRNA levels. We then performed immunoblot analysis to determine the size of the Vip3Aa11-m2 protein in VP2-142. Western blot results revealed that the Vip3Aa11-m2 protein was smaller compared to both the positive control and the Vip3Aa11-m1 protein in VP1-1 maize (**Figure 5B**). These findings strongly suggested that errors occurred during the translation process, resulting in the production of a truncated Vip3Aa11-m2 protein. This truncation is likely the primary cause of the observed loss of insecticidal activity in the Vip3Aa11-m2 protein.

### 3.6 The codon optimization of the 1 to 264 region in the *vip3Aa11-m2* gene resulted in a smaller Vip3Aa11-m2 protein

**Figure 6:**
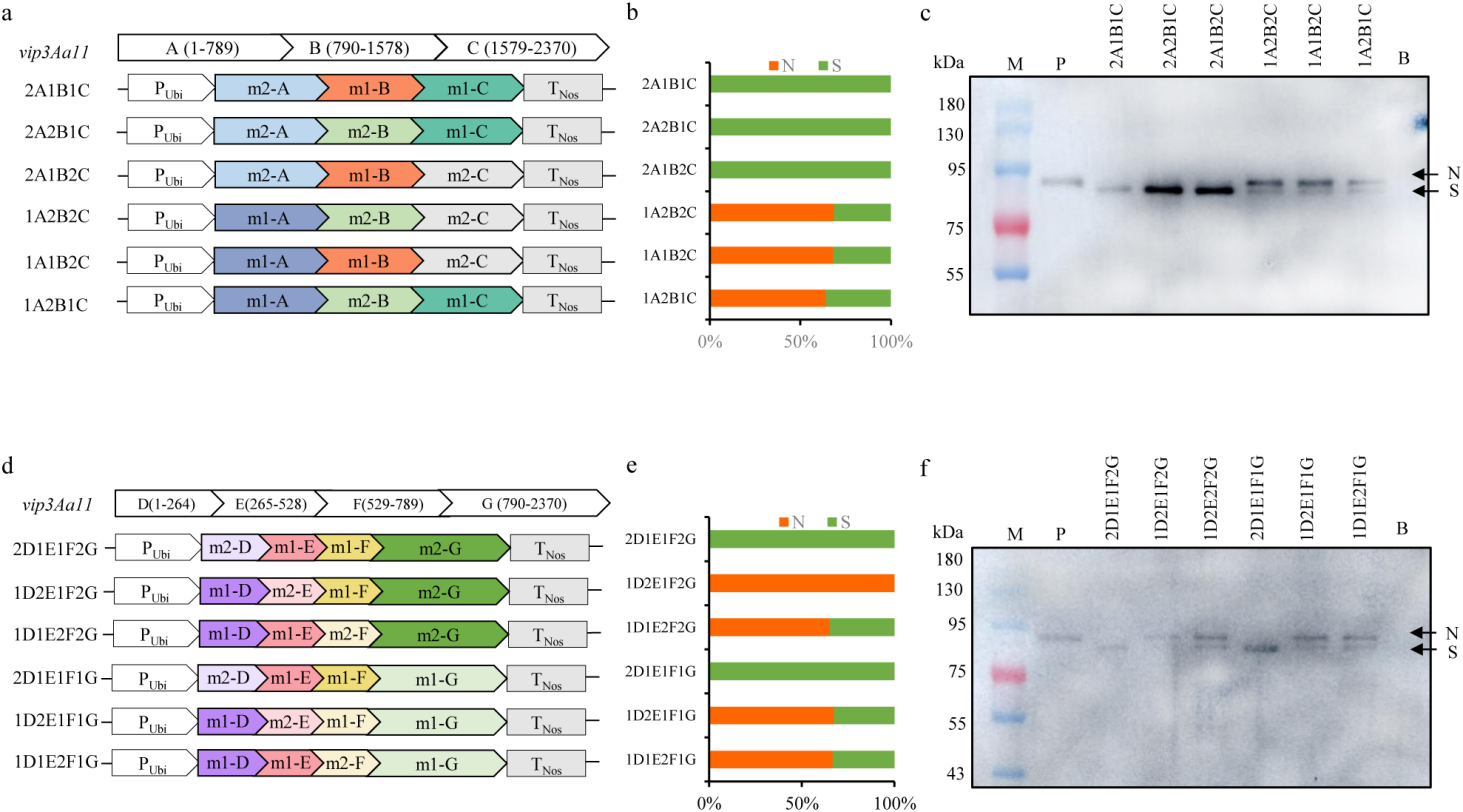
Immunoblot analysis of the size of recombinant Vip3Aa11 protein expressed in maize protoplasts. **(a)** Schematic diagram of the protoplasts transformation vectors. The *vip3Aa11* gene was divided into three segments: A (1-789), B (790-1578), and C (1579-2370). The different segments were substituted with those of *vip3Aa11-m1* and *vip3Aa11-m2* gene, resulting in six recombinant *vip3Aa11* gene expression vectors. **(b)** The ratio of normal size (N) Vip3Aa11 protein to small size (S) Vip3Aa11 protein expressed by six recombinant *vip3Aa11* genes, as shown in Figure **(c)** The calculation of the ratio was preformed using ImageJ 1.54g software. **(c)** Western blot analysis of the size of six recombinant Vip3Aa11 proteins (2A1B1C, 2A2B1C, 2A1B2C, 1A2B2C, 1A1B2C, and 1A2B1C) expressed in maize protoplasts. M: prestained protein ladder (10-180 kDa). P: Vip3Aa11-m1 protein expressed in VP1-1 transgenic maize plants was used as positive control; B: The empty vector was used as a blank control. **(d)** Schematic diagram of the protoplasts transformation vectors. The *vip3Aa11-*A segment was divided into three segments: D (1-264), E (265-528), and F (529-789). The different segments were substituted with those of *vip3Aa11-m1* and *vip3Aa11-m2* gene, resulting in six recombinant *vip3Aa11* gene expression vectors. **(e)** The ratio of normal size (N) Vip3Aa11 protein to small size (S) Vip3Aa11 protein expressed by six recombinant *vip3Aa11* genes, as shown in Figure **(f)** The calculation of the ratio was preformed using ImageJ 1.54g software. **(f)** Western blot analysis of the size of six recombinant Vip3Aa11 proteins (2D1E1F2G, 1D2E1F2G, 1D1E2F2G, 2D1E1F1G, 1D2E1F1G, and 1D1E2F1G) expressed in maize protoplasts. M: prestained protein ladder (10-180 kDa). P: Vip3Aa11-m1 protein expressed in VP1-1 transgenic maize plants was used as positive control; B: The empty vector was used as a blank control.

Due to the fact that the *vip3Aa11-m2* gene and the *vip3Aa11-m1* gene are two codon-optimized forms of the same gene, theoretically, the two proteins expressed should have been identical. However, the current result, where the Vip3Aa11-m2 protein was smaller than expected and has lost its insecticidal activity, can only be attributed to the optimized form of the *vip3Aa11-m2* gene. Nevertheless, it was remains unclear which specific region of the *vip3Aa11-m2* gene was responsible for this phenomenon. Therefore, a gene segmentation analysis was employed, whereby the *vip3Aa11-m2* gene was divided into three segments: m2-A (1 to 789), m2-B (790 to 1578), and m2-C (1579 to 2370), and the *vip3Aa11-m1* gene was similarly divided into the corresponding three segments. Subsequently, the different segments of m2 were substituted with those of m1, resulting in the construction of six expression vectors (**Figure 6A**). These six vectors were then transformed into maize protoplasts for recombinant protein expression, with the empty vector serving as a blank control. The immunoblotting results demonstrated that any vector containing the m2-A segment expressed a small-size protein (S) band compared to the Vip3Aa11-m1 protein, whereas vectors containing the m1-A segment expressed a normal-size protein (N) as the Vip3Aa11-m1 protein (**Figures 6B, C**). This suggested that the issue resided in the m2-A region. To more precisely locate the truncation-causing position, the m2-A segment was further divided into three sub-segments: m2-D (1 to 264), m2-E (265 to 528), and m2-F (529 to 789), and six additional expression vectors were constructed following the aforementioned procedure (**Figure 6D**). The immunoblotting result indicated that any vector containing the m2-D segment would express more small-size protein (S) than the other vectors containing the corresponding m1-D segment (**Figures 6E, F**). This finding suggests that the codon optimization of the 1 to 264 nucleotide region of *vip3Aa11-m2* gene is associated with protein processing and leads to the production of a smaller Vip3Aa11-m2 protein.

### 3.7 The codon of the N4 was crucial for the translation of the Vip3Aa11 protein

**Figure 7:**
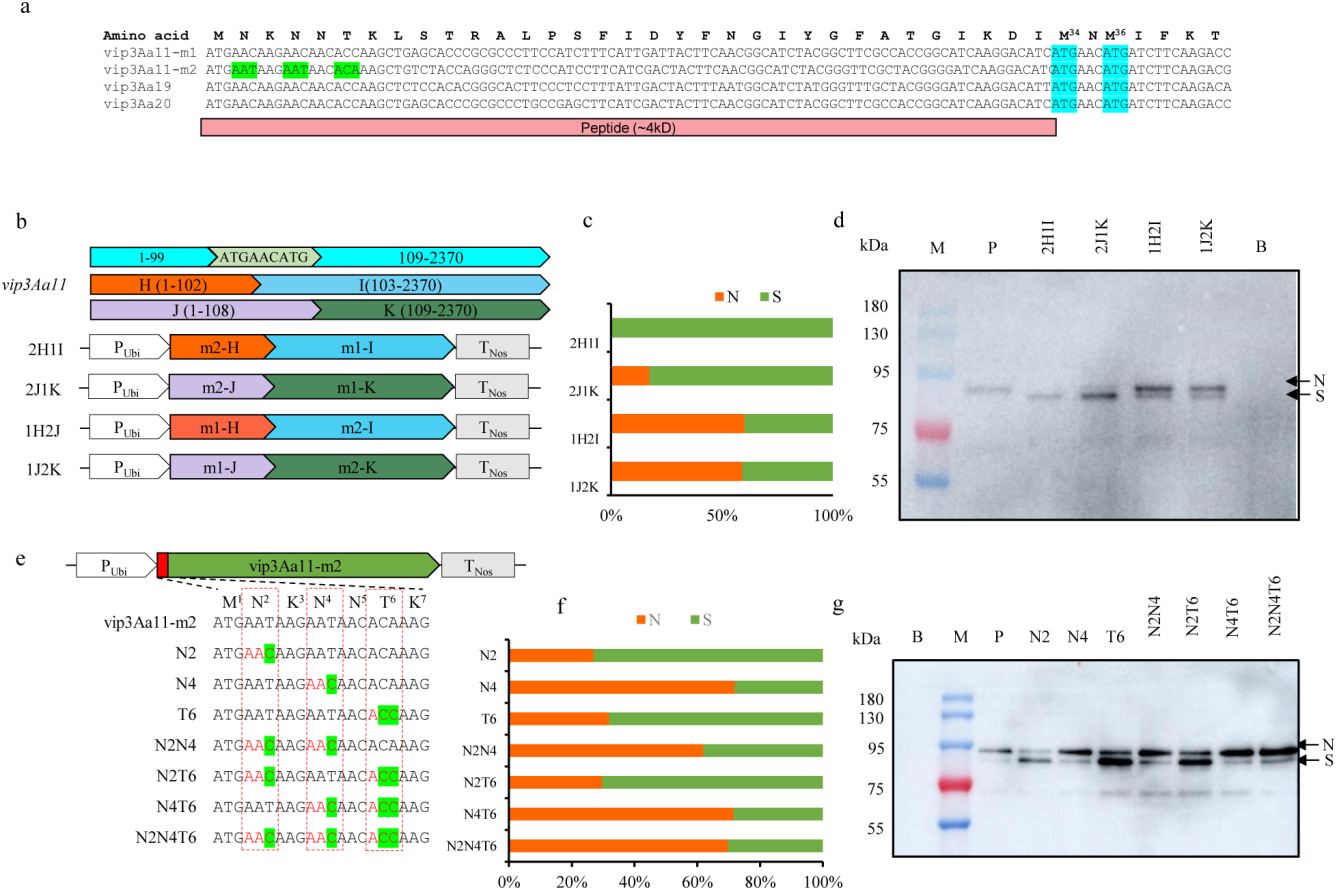
The codon of the N4 was crucial for the translation of the Vip3Aa11 protein. **(a)** Sequence alignment of the first 120 bp of the *vip3Aa11-m1*, *vip3Aa11-m2*, *vip3Aa19*, and *vip3Aa20* genes. The sequence of *vip3Aa19* and *vip3Aa20* genes were derived from the DBN9501 and MIR162 events, respectively. The codons that differ between *vip3Aa11-m2* and the other three genes (*vip3Aa11-m1*, *vip3Aa19*, and *vip3Aa20*) were highlighted in green. The codons of M34 and M36 were highlighted in blue. **(b)** Schematic diagram of the protoplasts transformation vectors. The first 102bp and 108bp of the *vip3Aa11* gene were substituted with those of *vip3Aa11-m1* and *vip3Aa11-m2* genes, resulting in four recombinant *vip3Aa11* gene expression vectors. **(c)** The ratio of normal size (N) Vip3Aa11 protein to small size (S) Vip3Aa11 protein expressed by four recombinant *vip3Aa11* genes, as shown in panel **(d). (d)** Western blot analysis of the size of four recombinant Vip3Aa11 proteins (2H1I, 2J1K, 1H2J, and 1J2K) expressed in maize protoplasts. M: prestained protein ladder (10-180 kDa). P: Vip3Aa11-m1 protein expressed in VP1-1 transgenic maize plants was used as positive control; B: The empty vector was used as a blank control. **(e)** Schematic diagram of the protoplasts transformation vectors. The codons N2, N4, and T6 in the *vip3Aa11-m2* gene were mutated to match those in the *vip3Aa11-m1* gene, resulting in the construction of seven expression vectors. **(f)** The ratio of normal size (N) Vip3Aa11 protein to small size (S) Vip3Aa11 protein expressed by seven mutated *vip3Aa11-m2* genes, as shown in panel **(g). (g)** Western blot analysis of the size of Vip3Aa11-m2 proteins (N2, N4, T6, N2N4, N2T6, N4T6 and N2N4T6) expressed in maize protoplasts. M: prestained protein ladder (10-180 kDa). P: Vip3Aa11-m1 protein expressed in maize protoplasts was used as positive control; B: The empty vector was used as a blank control.

There are usually two reasons for producing a smaller size protein than expected: one is the protein is cleaved after translation, and the other is the translation initiation site shift backward to next translation initiation site. Upon analyzing the 1 to 264 nucleotide region, two ATG codon encoding methionine were found at position 100-102 (M34) and 106-108 (M36) (**Figure 7A**). Therefore, the region spanning from 1 to 102 and the region from 1 to 108 of *vip3Aa11-m2* were designated as m2-H and m2-J respectively, for further segment replacement analysis. We replaced the m2-H and m2-J of the *vip3Aa11-m2* gene with those from the *vip3Aa11-m1 gene*, and vice versa, thus generating four expression vectors (**Figure 7B**). The western blot results demonstrated that when the m1-H or m1-J of *vip3Aa11-m1* were replaced with the corresponding sequence from *vip3Aa11-m2*, the majority of the produced Vip3Aa11 protein are of small size. However, when the m2-H or m2-J of *vip3Aa11-m2* were replaced with those from *vip3Aa11-m1*, the majority of the Vip3Aa11 protein returned to normal size (**Figures 7C, D**). By comparing the first 120 bps sequences of *vip3Aa11-m1*, *vip3Aa11-m2*, and the modified *vip3Aa19* and *vip3Aa20* genes, which have been proven to exhibit high insecticidal activity against FAW in transgenic maize (Wen et al., 2023; Zhang et al., 2024), we observed that only the *vip3Aa11-m2* gene had different synonymous codons at the 2nd amino acid N2 (AAT, Asn), the 4th amino acid N4 (AAT, Asn), and the 6th amino acid T6 (ACA, Thr) compared to the other three genes (**Figure 7A**). To identify which codon among the three amino acids in the *vip3Aa11-m2* gene plays a critical role in the translation of the Vip3Aa11-m2 protein, we mutated the codons of these amino acids to their corresponding forms in *vip3Aa11-m1*, thereby generating seven expression vectors (**Figure 7E**). Through mutation, we found that when the codon of N4 in the *vip3Aa11-m2* gene was changed from AAT to AAC, approximately 70% of the Vip3Aa11-m2 protein was restored to its normal size (**Figures 7F, G**). This indicated that the codon form of the 4th amino acid N4 is crucial for the translation of the Vip3Aa11 protein.

## 4 Discussion

### 4.1 N-terminus of Vip3Aa protein is essential for its insecticidal toxicity

The cryo-electron microscopy (cryo-EM) structure of the Vip3Aa toxin has revealed that the Vip3Aa protein is composed of five distinct domains (Nunez-Ramirez et al., 2020). Domain I extends from the N-terminus to the protease cleavage site and contains four α-helices (α1-α4). Domain II begins immediately after the cleavage site and comprises five α-helices (α5-α9) that form the core of the tetramer. Domain III encompasses three antiparallel β-sheets that form a β-prism fold strikingly similar to that found in members of the Cry insecticidal δ-endotoxins. Domain IV and domain V possess flexible carbohydrate-binding module (CBM) folds, which are connected to domain III by a long linker (**Supplementary Figure S1**). Two hypotheses have been proposed regarding the insecticidal mechanism of the Vip3Aa protein (Chakrabarty et al., 2020). The first hypothesis suggests that upon entering the target insect midgut, the Vip3Aa protein can directly bind to receptor on the surface of midgut cells without requiring any activation process. This binding may lead to receptor-mediated endocytosis, thereby allowing the Vip3Aa protein to enter the cells and induce apoptosis. The second hypothesis suggests that Vip3Aa protein is hydrolyzed by insect midgut proteases into two peptide fragments, with the 62–66 kDa fragment serving as the active center of the protein. This fragment can bind to receptors on the surface of midgut cells, forming pores in the cell membrane and ultimately resulting in cell death. However, recent findings have enhanced our understanding of the activation mechanism of Vip3Aa. Three-dimensional (3D) reconstruction has demonstrated that the Vip3Aa protoxin assembles into a pyramid-shaped tetramer, with the C-terminal domains exposed to the solvent and the N-terminal region folded into a spring-loaded apex. The α-helices within domain I undergo substantial reorganization, leading to the formation of a four-helix coiled coil upon trypsin treatment, which is of sufficient length to reach and permeate the lipid bilayer, thus forming pores in the insect cell (Nunez-Ramirez et al., 2020).

In previous studies, it has been found that the N-terminus of Vip3Aa protein plays a crucial role in its insecticidal toxicity. Specifically, the deletion of the first 198 amino acids from the N-terminus of Vip3Aa protein led to the loss of insecticidal activity against cotton bollworm and beet armyworm (Li et al., 2007a). Similarly, the removal of the first 27 amino acids of Vip3Aa reduced protein solubility and abolish insecticidal activity (Chen et al., 2003). Additionally, the absence of the first 39 amino acids of Vip3Aa decreased the virulence toward *Spodoptera litura* and *Chilo suppressalis*, respectively (Selvapandiyan et al., 2001). The 3D reconstruction also indicates that domain I of Vip3Aa is retained after protease digestion and remains tightly bound to the core of the protein through interactions with domain II, constituting an essential part of the active toxin (Nunez-Ramirez et al., 2020). It is thus reconfirmed that the N-terminus of the Vip3Aa protein is essential for its insecticidal toxicity. In this study, the truncated Vip3Aa11-m2 protein in VP2 transgenic maize, which lacks insecticidal activity, was attributed to the absence of the N-terminal ~33 amino acids, corresponding to a ~4 kDa peptide (**Figure 7A**). We are currently conducting N-terminal sequencing to confirm the translation initiation site of the truncated Vip3Aa11-m2 protein in VP2 transgenic maize.

### 4.2 Codon optimization can enhance protein expression

Bt insecticidal proteins, being safe biological insecticides, contribute to pest control and facilitate the management of Bt-resistant insect populations by virtue of their high expression and accumulation in plants. However, Bt toxin genes, derived from *Bacillus thuringiensis*, are AT-rich compared to the GC-rich plant genes. When Bt genes are directly transformed into plants, low expression in cells may occur due to multiple factors, including premature transcription termination, aberrant mRNA splicing, mRNA instability, or inefficient codon usage (Jabeen et al., 2010). Consequently, the production of effective levels of these Bt proteins in plants has necessitated the resynthesis of the encoding genes (Streatfield, 2007). Among the strategies employed to enhance the expression of exogenous genes in host plants, apart from utilizing strong promoters and enhancer elements, codon optimization is the most commonly used. The general consensus is that genes with a higher proportion of optimal codons are expressed more efficiently in a given organism (Webster et al., 2017). Genome-wide RNA decay analyses have shown that stable mRNAs are enriched in codons designated as optimal, whereas unstable mRNAs predominately contain non-optimal codons (Presnyak et al., 2015), providing robust support for this view. The strategies employed in codon optimization include: (1) optimizing codon sequences based on the host codon bias to increase the CAI value and enhance protein expression efficiency; (2) optimizing high GC content sequences by adjusting the GC content to an appropriate level to increase the probability of successful gene synthesis; (3) optimizing repetitive sequence regions to reduce the occurrence of repetitive sequences (Jackson et al., 2014). The use of codon optimization to enhance the expression of exogenous proteins in plants is widespread. For instance, the synthesized codon-modified *cry6A* gene exhibits high expression in tomato roots, providing resistance against root-knot nematodes (Li et al., 2007b). In our previous studies, we found that the codon-modified *cry1Ah* and *cry2Ah* genes significantly increased the protein expression in tobacco, conferring high resistance to cotton bollworm (Li et al., 2013, 2018). In the present study, the *vip3Aa11-m2* gene, which has a higher CAI, also led to higher protein expression in maize compared to the *vip3Aa11-m1* gene. Nevertheless, the gene expression process is complex, and optimal codon usage does not necessarily translate to the highest protein expression. For example, in tobacco, it has been observed that there is no increase in PAT protein expression when the percentage of optimal codons is increased from 63.9% to 93.9% (Agarwal et al., 2019). Sometimes, maintaining codon diversity while avoiding rare codons is more beneficial than always selecting preferred codons (Li et al., 2007b). Therefore, it is not necessary to aim for the highest proportion of optimal codons in the codon-modified gene.

### 4.3 Why the codon form of N4 can affect the translation of the Vip3Aa11 protein

Although numerous reports exist regarding the enhancement of protein expression through codon optimization, the outcomes in plants remain unpredictable (Jackson et al., 2014). In eukaryotes, the initiation of translation is a tightly regulated process that involves both *cis*-regulatory elements on the messenger RNA and trans-acting factors such as eukaryotic initiation factors (Tidu et al., 2024). During initiation, the eukaryotic 40S ribosomal subunit forms a 43S preinitiation complex (PIC) with eukaryotic initiation factors (eIFs) 1, 1A, 3, 5, and the eIF2•GTP•Met-tRNAi ternary complex (TC) (Wang et al., 2022). The scanning of the 43S PIC to select the start codon represents the most critical initial step in protein synthesis. Several studies suggest that the nucleotide context of the start codon plays a major role in start recognition efficiency (Kozak, 1986; Tidu and Martin, 2024). Based on these reports, a mechanism of translation initiation named STructure Assisted RNA Translation (START) has been proposed (Eriani and Martin, 2018; Despons and Martin, 2020). This mechanism implies that if the RNA secondary structure downstream of the start codon is stable enough to halt a scanning 43S PIC, translation efficiency is enhanced. Conversely, if the RNA secondary structure downstream of the start codon is insufficiently stable, the 43S PIC may perform leaky scanning of the start codon, resulting in incorrect translation. According to this mechanism, the results observed in this study can be attributed to the change in the 4th amino acid codon N4 of the Vip3Aa11 protein. This change may lead to the instability of the downstream RNA secondary structure, thereby causing N-terminal truncation during protein translation. Besides START mechanism, protein modification likely destabilizes the downstream RNA secondary structure, leading to N-terminal truncation during translation. Supporting this mechanism, N4-acetylcytidine and similar protein modifications have been demonstrated to affect mRNA secondary structure, consequently altering ribosome binding and scanning efficiency - as observed both in HeLa cells and *in vitro* systems (Arango et al., 2022). Additionally, the structural insights into the mechanism of ribosome scanning and start codon recognition highlight the critical role of mRNA secondary structure in translation initiation (Hinnebusch, 2017). Although the precise reason for the reduction in size of the Vip3Aa11 protein resulting from AAC to AAT in the codon for the 4th amino acid (Asn) remains elusive, our study demonstrates that the N4 codon, which is AAC, plays a crucial role in maintaining the integrity of the Vip3Aa11 protein and its insecticidal activity. Therefore, utilizing the *vip3Aa11* gene to developing insect-resistant crops in the future, alteration of this site should be avoided.

## Data Availability

The datasets presented in this study can be found in online repositories. The names of the repository/repositories and accession number(s) can be found in the article/**Supplementary Material**.
