# Supplementary material for: Impact of codon optimization on vip3Aa11 gene expression and insecticidal efficacy in maize
Source: Front Plant Sci. 2025 May 13;16:1579465. doi: 10.3389/fpls.2025.1579465 (PMC12106571; doi:10.3389/fpls.2025.1579465)
Supplement: Supplementary file 1 [file Table1.docx]

Supplementary Material

# Supplementary Figures and Tables

## Supplementary Figures


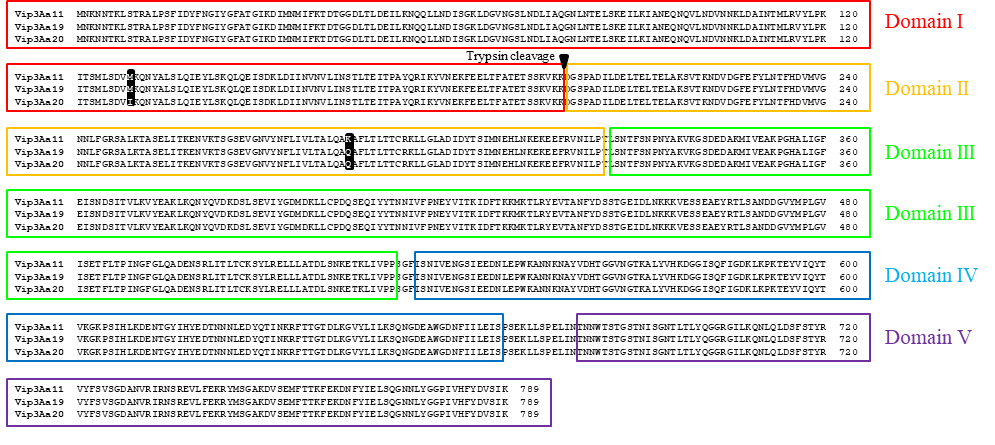


**Supplementary Figure 1.** Protein sequence alignment of Vip3Aa11, Vip3Aa19, Vip3Aa20 and their structures. The protein sequences used are as follows: Vip3Aa11 (GenBank accession number AAR36859), Vip3Aa19 (DQ539887), and Vip3Aa20 (DQ539888). Domains are highlighted in colored boxes.

## Supplementary Tables

**Supplementary Table 1.** Primers used in this study.

| Primer name | Sequence | Purpose |
| --- | --- | --- |
| vip3Aa11-m1-F1 | 5'-AAATCACTCCCGCCTACCAA-3' | PCR primers for detection of *vip3Aa11-m1* gene |
| vip3Aa11-m1-R1 | 5'-TGATTGGGGTCAGGAAGGTC-3' |  |
| cp4-F1 | 5'-TCGTCGTGGGGATTGAAGAA-3' | PCR primers for detection of *cp4-epsps* gene |
| cp4-R1 | 5'-GGTTCATCAGCACGTTGAGG-3' |  |
| vip3Aa11-m2-F1 | 5'-CGTGTTCCCCAACGAGT-3' | PCR primers for detection of *vip3Aa11-m2* gene |
| vip3Aa11-m2-R1 | 5'-TGCTTCAGGATGCCCC-3' |  |
| bar-F1 | 5'-TGACGCACAATCCCACTATCCT-3' | PCR primers for detection of *bar* gene |
| bar-R1 | 5'-CAGCGACCACGCTCTTGAAGC-3' |  |
| vip3Aa11-m1-F2 | 5'-ATGAACAAGAACAACACCAAGCT-3' | Primers for amplification of full-length *vip3Aa11-m1* gene |
| vip3Aa11-m1-R2 | 5'-TCACTTAATGCTAACGTCGTAAAAGT-3' |  |
| vip3Aa11-m2-F2 | 5'-ATGAATAAGAATAACACAAAGCTGTCT-3' | Primers for amplification of full-length *vip3Aa11-m2* gene |
| vip3Aa11-m2-R2 | 5'-TCACTTGATGGACACGTCGT-3' |  |
| m1-A-F1 | 5'-ATGAACAAGAACAACACCAA-3' | Primers for amplification of *vip3Aa11-m1-A* segment |
| m1-A-R1 | 5'-GGTCTTCACGTTTTCCTTGG-3' |  |
| m1-B-F1 | 5'-AGCGGCAGCGAGGTCGGCAA-3' | Primers for amplification of *vip3Aa11-m1-B* segment |
| m1-B-R1 | 5'-CTTAGTCTCCTTGTTGGACA-3' |  |
| m1-C-F1 | 5'-CTGATCGTCCCCCCATCGGG-3' | Primers for amplification of *vip3Aa11-m1-C* segment |
| m1-C-R1 | 5'-TCACTTAATGCTAACGTCGT-3' |  |
| m2-A-F1 | 5'-ATGAATAAGAATAACACAAA-3' | Primers for amplification of *vip3Aa11-m2-A* segment |
| m2-A-R1 | 5'-CGTCTTCACGTTCTCCTTGG-3' |  |
| m2-B-F1 | 5'-TCCGGCAGCGAAGTGGGCAA-3' | Primers for amplification of *vip3Aa11-m2-B* segment |
| m2-B-R1 | 5'-CTTCGTCTCCTTGTTGCTCA-3' |  |
| m2-C-F1 | 5'-CTCATCGTGCCGCCCTCCGG-3' | Primers for amplification of *vip3Aa11-m2-C* segment |
| m2-C-R1 | 5'-TCACTTGATGGACACGTCGT-3' |  |
| m1-A-F1 | 5'-ATGAACAAGAACAACACCAA-3' | Primers for amplification of *vip3Aa11-m1-D* segment |
| m1-D-R1 | 5'-CTCCTTGCTCAGTTCCGTGT-3' |  |
| m1-E-F1 | 5'-ATCCTCAAGATTGCTAACGA-3' | Primers for amplification of *vip3Aa11-m1-E* segment |
| m1-E-R1 | 5'-GATACGTTGGTAGGCGGGAG-3' |  |
| m1-F-F1 | 5'-AAGTACGTTAACGAAAAGTT-3' | Primers for amplification of *vip3Aa11-m1-F* segment |
| m1-A-R1 | 5'-GGTCTTCACGTTTTCCTTGG-3' |  |
| m1-B-F1 | 5'-AGCGGCAGCGAGGTCGGCAA-3' | Primers for amplification of *vip3Aa11-m1-G* segment |
| m1-C-R1 | 5'-TCACTTAATGCTAACGTCGT-3' |  |
| m2-A-F1 | 5'-ATGAATAAGAATAACACAAA-3' | Primers for amplification of *vip3Aa11-m2-D* segment |
| m2-D-R1 | 5'-CTCCTTGCTCAGCTCGGTGT-3' |  |
| m2-E-F1 | 5'-ATCCTCAAGATCGCGAACGA-3' | Primers for amplification of *vip3Aa11-m2-E* segment |
| m2-E-R1 | 5'-GATGCGCTGGTACGCCGGGG-3' |  |
| m2-F-F1 | 5'-AAGTATGTGAACGAGAAGTT-3' | Primers for amplification of *vip3Aa11-m2-F* segment |
| m2-A-R1 | 5'-CGTCTTCACGTTCTCCTTGG-3' |  |
| m2-B-F1 | 5'-TCCGGCAGCGAAGTGGGCAA-3' | Primers for amplification of *vip3Aa11-m2-G* segment |
| m2-C-R1 | 5'-TCACTTGATGGACACGTCGT-3' |  |
| m1-A-F1 | 5'-ATGAACAAGAACAACACCAA-3' | Primers for amplification of *vip3Aa11-m1-H* segment |
| m1-H-R1 | 5'-CATGATGTCCTTGATGCCGG-3' |  |
| m1-I-F1 | 5'-AACATGATCTTCAAGACCGA-3' | Primers for amplification of *vip3Aa11-m1-I* segment |
| m1-C-R1 | 5'-TCACTTAATGCTAACGTCGT-3' |  |
| m2-A-F1 | 5'-ATGAATAAGAATAACACAAA-3' | Primers for amplification of *vip3Aa11-m2-H* segment |
| m2-H-R1 | 5'-CATGATGTCCTTGATCCCCG-3' |  |
| m2-I-F1 | 5'-AACATGATCTTCAAGACGGA-3' | Primers for amplification of *vip3Aa11-m2-I* segment |
| m2-C-R1 | 5'-TCACTTGATGGACACGTCGT-3' |  |
| m1-A-F1 | 5'-ATGAACAAGAACAACACCAA-3' | Primers for amplification of *vip3Aa11-m1-J* segment |
| m1-J-R1 | 5'-CATGTTCATGATGTCCTTGA-3' |  |
| m1-K-F1 | 5'-ATCTTCAAGACCGACACCGG-3' | Primers for amplification of *vip3Aa11-m1-K* segment |
| m1-C-R1 | 5'-TCACTTAATGCTAACGTCGT-3' |  |
| m2-A-F1 | 5'-ATGAATAAGAATAACACAAA-3' | Primers for amplification of *vip3Aa11-m2-J* segment |
| m2-J-R1 | 5'-CATGTTCATGATGTCCTTGA-3' |  |
| m2-K-F1 | 5'-ATCTTCAAGACGGACACGGG-3' | Primers for amplification of *vip3Aa11-m2-K* segment |
| m2-C-R1 | 5'-TCACTTGATGGACACGTCGT-3' |  |
| N2-F1 | 5'-ATGAACAAGAATAACACAAAG-3' | Primers for amplification of N2, N4, T6, N2N4, N2T6, N4T6 and N2N4T6 mutants, respectively |
| N4-F1 | 5'-ATGAATAAGAACAACACAAAG-3' |  |
| T6-F1 | 5'-ATGAATAAGAATAACACCAAG-3' |  |
| N2N4-F1 | 5'-ATGAACAAGAACAACACAAAG-3' |  |
| N2T6-F1 | 5'-ATGAACAAGAATAACACCAAG-3' |  |
| N4T6-F1 | 5'-ATGAATAAGAACAACACCAAG-3' |  |
| N2N4T6-F1 | 5'-ATGAACAAGAACAACACCAAG-3' |  |
| m2-C-R1 | 5'-TCACTTGATGGACACGTCGT-3' |  |

**Supplementary Table 2.** The mortality rates of FAW larvae after 5 days of feeding on the leaves of transgenic maize plants.

| Transgenic lines^a^ | Vip3Aa11 expression^b^ (μg/g fresh weight) | Mortality rates (%) of fall armyworm^c^ |
| --- | --- | --- |
| VP1-1 | 1.58±0.04 | 100.00±0.00a |
| VP1-6 | 1.50±0.07 | 100.00±0.00a |
| VP1-13 | 1.59±0.07 | 100.00±0.00a |
| VP1-15 | 1.19±0.09 | 100.00±0.00a |
| VP1-18 | 1.33±0.06 | 100.00±0.00a |
| VP1-21 | 1.87±0.07 | 100.00±0.00a |
| VP1-22 | 1.43±0.06 | 100.00±0.00a |
| VP1-24 | 1.58±0.04 | 100.00±0.00a |
| VP1-31 | 2.48±0.09 | 100.00±0.00a |
| VP1-77 | 2.29±0.05 | 100.00±0.00a |
| VP1-81 | 2.76±0.12 | 100.00±0.00a |
| VP1-82 | 1.37±0.04 | 100.00±0.00a |
| VP1-83 | 1.17±0.05 | 100.00±0.00a |
| VP1-84 | 1.35±0.06 | 100.00±0.00a |
| VP1-88 | 1.68±0.03 | 100.00±0.00a |
| VP1-95 | 1.96±0.08 | 100.00±0.00a |
| VP1-96 | 2.02±0.07 | 100.00±0.00a |
| VP1-101 | 1.47±0.04 | 100.00±0.00a |
| VP1-113 | 1.38±0.04 | 100.00±0.00a |
| VP1-118 | 1.38±0.08 | 100.00±0.00a |
| VP1-120 | 1.57±0.04 | 100.00±0.00a |
| VP1-122 | 1.47±0.09 | 100.00±0.00a |
| VP1-134 | 1.28±0.03 | 100.00±0.00a |
| VP2-2 | 2.44±0.11 | 11.11±4.81b |
| VP2-3 | 4.24±0.20 | 9.72±6.36b |
| VP2-4 | 3.07±0.08 | 8.33±0.00b |
| VP2-6 | 4.44±0.16 | 5.56±9.62b |
| VP2-7 | 1.77±0.07 | 9.72±2.41b |
| VP2-8 | 1.23±0.07 | 8.33±7.22b |
| VP2-13 | 3.25±0.09 | 9.72±8.67b |
| VP2-17 | 1.66±0.08 | 9.72±6.36b |
| VP2-18 | 6.38±0.44 | 8.33±7.22b |
| VP2-21 | 2.94±0.09 | 4.17±7.22b |
| VP2-24 | 1.17±0.04 | 13.89±4.81b |
| VP2-26 | 2.33±0.12 | 5.56±4.81b |
| VP2-27 | 2.98±0.05 | 5.56±9.62b |
| VP2-29 | 3.64±0.30 | 9.72±8.67b |
| VP2-31 | 1.26±0.05 | 9.72±8.67b |
| VP2-32 | 3.11±0.20 | 11.11±2.41b |
| VP2-35 | 2.57±0.19 | 11.11±4.81b |
| VP2-38 | 3.57±0.22 | 11.11±6.36b |
| VP2-42 | 4.82±0.14 | 5.56±9.62b |
| VP2-43 | 7.09±0.20 | 9.72±6.36b |
| VP2-52 | 3.47±0.09 | 12.50±4.17b |
| VP2-57 | 2.03±0.12 | 8.33±0.00b |
| VP2-58 | 3.55±0.06 | 5.56±6.36b |
| VP2-64 | 9.91±0.12 | 9.72±8.67b |
| VP2-65 | 3.88±0.15 | 9.72±4.81b |
| VP2-67 | 3.37±0.20 | 6.94±8.67b |
| VP2-71 | 3.45±0.20 | 4.17±4.17b |
| VP2-72 | 2.94±0.11 | 4.17±4.17b |
| VP2-82 | 3.94±0.09 | 8.33±4.17b |
| VP2-83 | 3.53±0.04 | 11.11±9.62b |
| VP2-87 | 4.68±0.17 | 15.28±2.41b |
| VP2-92 | 1.39±0.10 | 12.50±7.22b |
| VP2-94 | 4.09±0.13 | 9.72±6.36b |
| VP2-95 | 8.72±0.23 | 15.28±2.41b |
| VP2-101 | 10.09±0.13 | 11.11±9.62b |
| VP2-104 | 7.01±0.15 | 4.17±0.00b |
| VP2-113 | 11.77±0.12 | 9.72±6.36b |
| VP2-114 | 4.17±0.08 | 11.11±4.81b |
| VP2-118 | 3.07±0.08 | 12.50±0.00b |
| VP2-121 | 3.87±0.07 | 8.33±7.22b |
| VP2-125 | 3.85±0.13 | 4.17±7.22b |
| VP2-126 | 11.01±0.21 | 9.72±4.81b |
| VP2-131 | 10.71±0.13 | 4.17±4.17b |
| VP2-136 | 2.64±0.13 | 11.11±6.36b |
| VP2-138 | 1.37±0.09 | 5.56±9.62b |
| VP2-139 | 4.80±0.18 | 8.33±7.22b |
| VP2-142 | 11.45±0.43 | 6.94±8.67b |
| VP2-143 | 1.97±0.07 | 12.50±7.22b |
| VP2-144 | 1.86±0.09 | 8.33±7.22b |
| B104 | ND | 4.17±4.17b |

^a^VP1 and VP2 represent vip3Aa11-m1 and vip3Aa11-m2 transgenic maize plants, respectively.

^b^Vip3Aa11 protein expression levels in transgenic maize plants. Data represent means±SD (n=3 technical replicates).

^c^The proportion of dead larvae to total larvae applied (%) of feeding on the leaves of transgenic maize plants. Data represent means±SD (n=3 biological replicates).
